# Recovery of ferulic acid from nixtamalization wastewater by adsorption on MIL-88D(Fe)-MOF

**DOI:** 10.1039/d6ra06031e

**Published:** 2026-10-12

**Authors:** W. Y. Padrón-Hernández, L. M. Cervera-Andrade, A. P. M. Hernández-Muñoz, D. D. Cabañas-Vargas, E. G. Uc-Cayetano, G. Rodríguez-Gattorno, M. N. Sánchez-González, M. A. Estrella-Gutiérrez

**Affiliations:** a Facultad de Ingeniería Química, Universidad Autónoma de Yucatán Periférico Norte, Kilómetro 33.5. Tablaje Catastral 13615, Chuburná de Hidalgo Inn, C.P. 97203 Mérida Yucatán Mexico monica.sanchez@correo.uady.mx alejandro.estrella@correo.uady.mx; b Departamento de Física Aplicada, Centro de Investigación y de Estudios Avanzados Del Instituto Politécnico Nacional Unidad Mérida, Km 6 Carretera Antigua a Progreso, Cordemex Mérida 97310 Yucatán Mexico

## Abstract

Ferulic acid (FA) is a high-value phenolic compound present in nixtamalization wastewater (nejayote), whose recovery could contribute to the valorization of this agro-industrial effluent. In this work, a microwave-synthesized flexible iron-based metal–organic framework, MIL-88D(Fe), was evaluated for FA adsorption and recovery from model solutions and real nejayote. Nonlinear kinetic analysis showed that the Elovich model provided the best statistical description of FA uptake, closely followed by the pseudo-second-order model, while the Freundlich isotherm best described the adsorption data at 311 K. The highest experimentally measured adsorption capacity was 30.36 mg g^−1^ at 311 K, and FA removal reached 91.62% at pH 3.3 in model solutions. In real nejayote, MIL-88D(Fe) removed 90.34% of FA and enabled an overall FA recovery of 74.19% after alkaline desorption. Reusability tests showed removal efficiencies of 87.19–75.19% over five consecutive adsorption–desorption cycles, corresponding to retention of 86.24% of the first-cycle performance. XRD and SEM analyses after regeneration confirmed preservation of crystallinity and rod-like morphology. These results demonstrate that MIL-88D(Fe) combines effective FA capture, recoverability, and recyclability, supporting its potential as a reusable adsorbent for recovery-oriented treatment and valorization of nixtamalization wastewater.

## Abstract 2

MIL-88D(Fe) enables selective recovery of ferulic acid from real nejayote, combining fast adsorption, 91.62% removal in model solutions, and wastewater valorization into valuable phenolic compounds.

## Graphical abstract

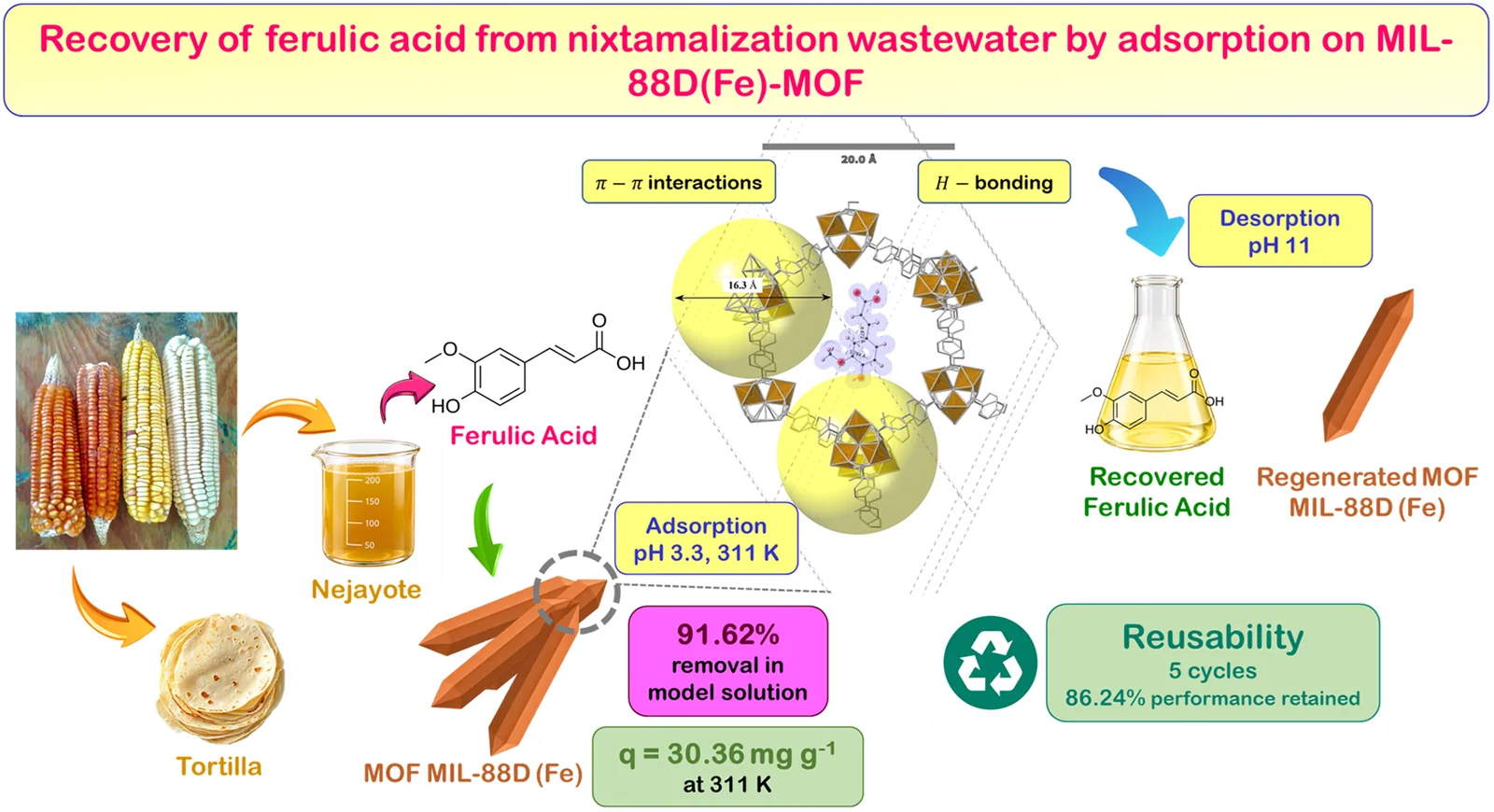

## 1. Introduction

Ferulic acid (FA), 4-hydroxy-3-methoxycinnamic acid, is a ubiquitous derivative of cinnamic acid. It is primarily covalently bound within the cell wall, crosslinking polysaccharides and lignin, thereby providing rigidity, strength and structural stability. FA is abundant in cereals, grains, fruits and vegetables, with maize being the cereal that contains the highest concentration of FA.^1^

FA possesses a wide range of physiological functions. It is a potent antioxidant and is widely used in the food, cosmetic, and pharmaceutical industries due to its biological activities, including anti-inflammatory, antimicrobial, anticancer, and antidiabetic effects.^1–3^

Maize is a staple food in Mexico, mainly consumed as tortillas (thin, round, unleavened breads). For tortilla preparation, maize is cooked under alkaline conditions, through a process known as nixtamalization.^4^ This process partially solubilizes maize cell wall components, including FA, into the alkaline wastewater obtained, known as nejayote.^5^ The high daily production of tortillas in Mexico (approximately 32000 tons) generates around 14.4 million cubic meters of nejayote.^4,6,7^ Currently, this residual water is either treated of or discharged into sewage systems; however, it contains significant amounts of FA.^8,9^ Although alternative uses for nejayote are being explored, much of it is still discarded, highlighting the need for efficient valorization strategies^8,9^

The recovery of FA from such complex matrices (high alkalinity, organic load) requires selective and efficient methods. Adsorption techniques have emerged as promising approaches, and recent advances in porous materials have opened new opportunities in this field. Metal–Organic Frameworks (MOFs) are a class of hybrid porous materials composed of metal ions or clusters coordinated with organic linkers. Their high surface area, tunable porosity, and functionalizable surfaces make them exceptional adsorbents for various applications, including gas storage, catalysis, and the removal of heavy metals or organic pollutants.^10^

Recent studies have demonstrated the potential of specific MOFs for the adsorption and recovery of phenolic compounds, including FA, from aqueous solutions.^11,12^ While various metals (*e.g.*, Al, Cr, Zr) and MOF families (*e.g.*, UiO-66, MIL-101) could potentially be used, iron-based MOFs offer a highly compelling platform. These MOFs are notable for their high safety, biodegradability, and strong chemical stability in both organic solvents and water due to the robust coordination between Fe and O atoms. The primary advantage of iron over other metals is its ability to successfully balance chemical stability with biosafety, a challenge where alternatives often fail. Iron is the fourth most abundant metal in the Earth's crust, an essential trace element for humans, and environmentally benign. As a commercially available and nontoxic material, it is considered the least hazardous metal used in MOF synthesis. This safety profile is particularly advantageous for applications involving food grade or nutraceutical compound recovery, as it minimizes concerns regarding metal leaching and sample matrix contamination.^13–16^

Within this category, the iron-based MIL-88 family is of particular interest due to its structural flexibility, which provides a controllable pore size and internal environment that facilitates the entry of guest molecules. Specifically, MIL-88D(Fe), constructed from iron(iii) clusters and biphenyl-4,4′-dicarboxylate (BPDC) linkers, presents an optimal architecture. The pore dimensions, dictated by the relatively long BPDC linker, are highly suitable for accommodating target molecules like FA. Ultimately, the iron-based MIL-88D offers a highly favorable combination of safety, structural adaptability, and chemical stability in aqueous media, making it an ideal candidate for the efficient recovery of FA.

This work investigates the application of the MIL-88D(Fe)-MOF for the adsorption and recovery of FA, first from aqueous model solutions and subsequently from commercial nejayote samples. Despite prior use of adsorbents and MOFs for the removal of phenolic compounds, most studies have focused on model aqueous systems and have not addressed adsorption–desorption cycles in real agro-industrial wastewater. The study comprehensively evaluates the synthesis and characterization of the adsorbent, its adsorption kinetics, isotherm equilibrium, and the effects of critical parameters such as pH. Validation in a real matrix and a mechanistic discussion of adsorption behavior allowed the development of an efficient and sustainable strategy for valorizing nixtamalization wastewater, transforming environmental liability into a source of valuable biochemicals.

## 2. Experimental

### 2.1. Materials

Ferric nitrate nonahydrate (Fe(NO_3_)_3_·9H_2_O, 98%), biphenyl-4,4′-dicarboxylic acid (BPDC, 97%), diethylformamide (DEF, 99%), *N*,*N*-dimethylformamide (DMF, C_3_H_7_ON, 99.8% anhydrous), dichloromethane (DCM, 99.8%) and isopropanol (70%) were obtained from Sigma-Aldrich and were used for MOF synthesis. FA (99%) standard was obtained from Sigma-Aldrich in powdered form. In addition, sodium carbonate (99%), hydrochloric acid (36%), and Folin–Ciocalteu's reagent were supplied from Sigma-Aldrich and were used for FA determination. In addition, for HPLC analysis, water, methyl alcohol and acetonitrile were used for the quantification of FA. All chemicals were used without further purification.

### 2.2. Synthesis of MIL-88D (Fe)-MOF

1 mmol of BPDC was suspended in 50 mL of DEF and sonicated for 10 min in an ultrasonic bath. Subsequently, 2 mmol of ferric nitrate nonahydrate (Fe(NO_3_)_3_·9H_2_O) was added to the mixture. The synthesis was carried out in a CEM MARS 6 microwave reactor using a 100 mL Teflon-lined high-pressure vessel (EasyPrep Plus). The reaction was run in temperature-control mode at a maximum power of 800 W with continuous magnetic stirring. While the pressure was monitored, it was not actively controlled, reaching a maximum of approximately 10.3 bar. The temperature program consisted of a 5 minute ramp to 373 K, followed by a 30 minute hold at that temperature.

Finally, the microwave reactor went through a cooling stage for 15 min until reaching a temperature of 298 K. After MOF synthesis, the material was centrifuged at 4500 rpm for 10 min and then suspended in 10 mL of DMF for 24 h with stirring. This washing process was repeated for two additional days, alternating centrifugation cycles at 4500 rpm for 10 min between each washing step. Additional washes were then carried out for three consecutive days using 10 mL of isopropanol, followed by one day of washing with 10 mL of dichloromethane. On each of these days, centrifugation cycles were alternated at 4500 rpm for 10 min to ensure adequate cleaning of the material. Finally, the purified MOF was dried under vacuum overnight at 333 K to remove any remaining solvent residue and ensure a dry and stable product.

### 2.3. Characterization

To verify the crystallinity of the samples, powder X-ray diffraction analysis was performed using a Bruker D-8 Advance at 40 kV and 30 mA for Cu-Kα (*λ* = 1.5418°) in the range of 0° to 20° 2*θ* degrees with a speed of 1° per minute with a step interval of 0.02°. The morphology of the MOF powder was examined by scanning electron microscopy using a JEOL JSM-7600F. The samples were covered with a layer of Au–Pd for 40 seconds using an acceleration voltage of 5 kV. FTIR analysis was conducted on the Agilent Cary 630 FTIR spectrometer using the ATR (Attenuated Total Reflection) technique to identify the functional groups present in the materials. The functional groups were analyzed within a 4000 to 500 cm^−1^ wavelength range. Gas adsorption measurements were carried out with N_2_ at 77 K with a surface area and porosity analyzer (BELSORP-Max LP) after activation of samples at 393 K for 2 h. Surface area and total pore volume were obtained using BET method.

### 2.4. Adsorption kinetics

A small working volume of 1 mL was used to minimize the consumption of the synthesized MOF material while maintaining a quantifiable concentration for accurate spectrophotometric analysis. Experiments were performed adding 1 mL of FA (1 g L^−1^) to 5, 10, 15, and 30 mg of MIL-88D (Fe)-MOF. Reactions were incubated at 311 K, 100 rpm, for 100 min, taking a sample every 20 min. The reactions were stopped by centrifugation at 4500 rpm and the amount of residual FA in the solution was determined by the Folin–Ciocalteu colorimetric method.^17^

The FA removal (%) was calculated using eqn (1) and (2):1
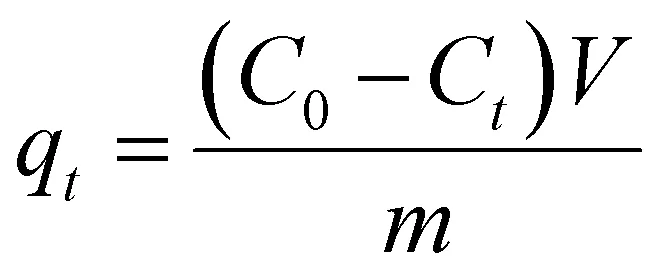
2
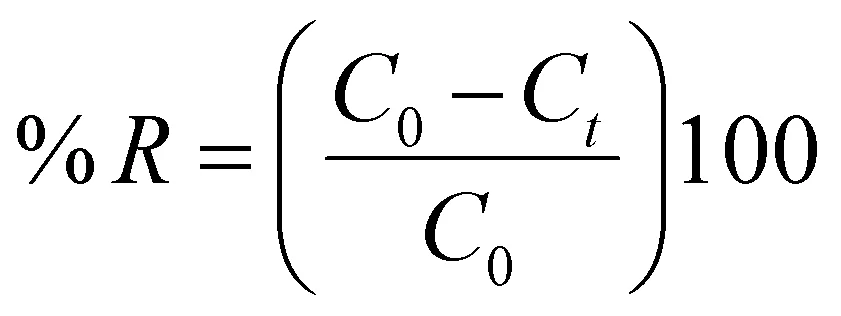
where *C*_0_, *C*_*t*_ (mg L^−1^) represent FA concentration at zero and *t* time, respectively. *V* (L) is the volume of FA solution and *m* (g) is the amount of MOF MIL-88D (Fe). The adsorption capacity (*q*_*t*_) is expressed in mg g^−1^.

The experimental kinetic data were analyzed by direct nonlinear regression using the pseudo-first-order (PFO), pseudo-second-order (PSO), and Elovich kinetic models. Direct fitting of the original *q*_*t*_*versus t* data was selected instead of fitting linearized forms because mathematical transformation of nonlinear kinetic equations may modify the experimental error structure and result in biased parameter estimates.^18–20^

The nonlinear pseudo-first-order model was expressed as:

3 *q*_*t*_ = *q*_e_(1 − e^−*k*_1_*t*^)

where *q*_e_ is the model-estimated adsorption capacity at equilibrium (mg g^−1^) and *k*_1_ is the pseudo-first-order rate constant (min^−1^).

The nonlinear pseudo-second-order model was expressed as:4
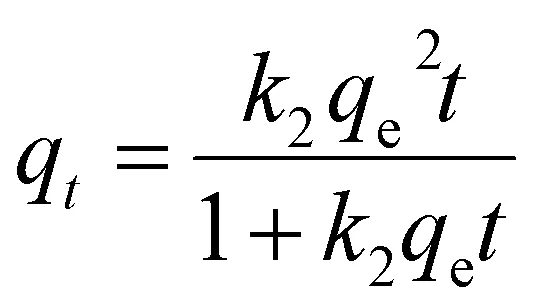
where *k*_2_ is the pseudo-second-order rate constant (g mg^−1^ min^−1^).

The Elovich model was described by:5
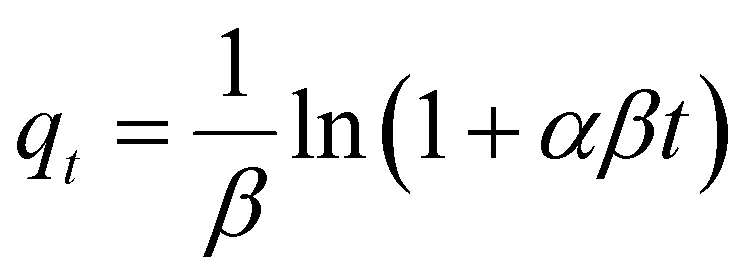
where *α* is the initial adsorption-rate parameter (mg g^−1^ min^−1^), and *β* is the Elovich parameter (g mg^−1^), commonly associated with the decrease in adsorption rate with increasing surface coverage.

In addition to the nonlinear kinetic models, the possible contribution of intraparticle mass transfer was evaluated using the Weber–Morris intraparticle diffusion (IPD) relationship:

6 *q*_*t*_ = *k*_id_*t*^1/2^ + *C*

where *k*_id_ is the intraparticle diffusion rate constant (mg g^−1^ min^−1/2^) and *C* is the intercept (mg g^−1^) and is related to the boundary-layer contribution. The Weber–Morris analysis was performed from the relationship between *q*_*t*_ and *t*^1/2^. Because this expression is linear with respect to *t*^1/2^, it was evaluated separately from the direct nonlinear PFO, PSO, and Elovich regressions.

Model parameters for the PFO, PSO, and Elovich equations were estimated by nonlinear least-squares regression through minimization of the sum of squared residuals between experimental and model-calculated adsorption capacities. The goodness of fit was evaluated using the coefficient of determination (*R*^2^), the sum of squared errors (SSE), and the root-mean-square error (RMSE).^20^

The SSE was calculated as:7
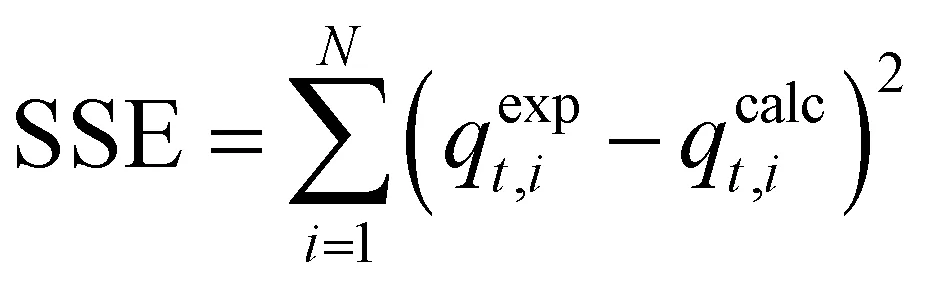
the RMSE as:8
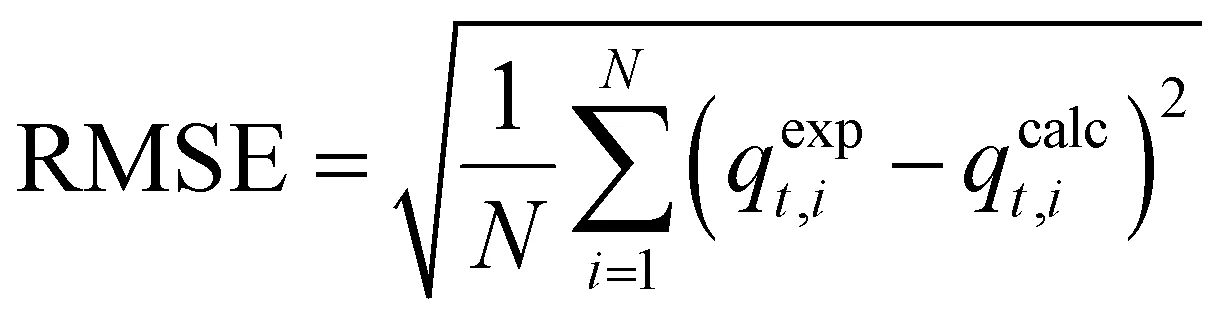
and the coefficient of determination as:9
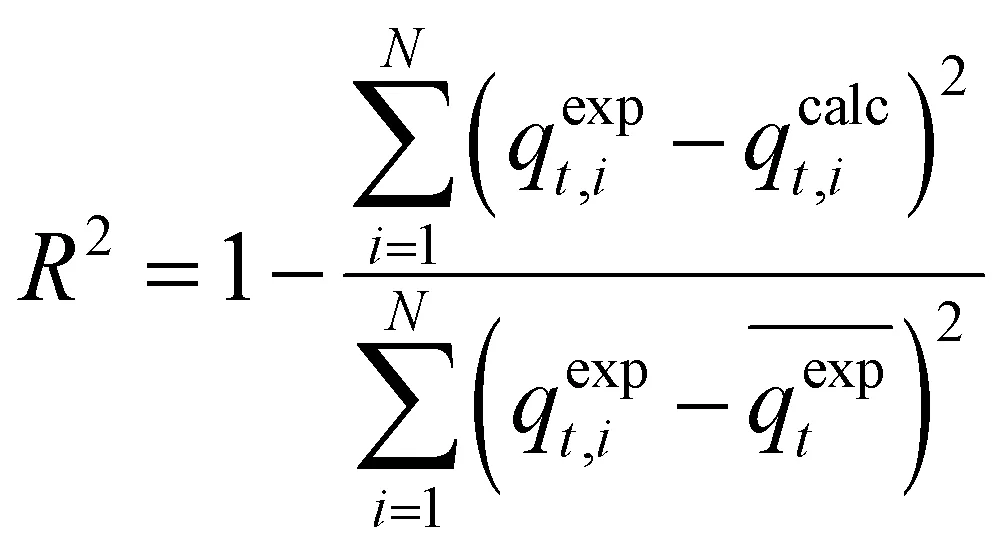
where *q*^exp^_*t*,*i*_ and *q*^calc^_*t*,*i*_ are the experimental and model-calculated adsorption capacities for the *i*-th observation, respectively; 
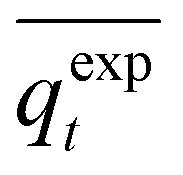
 is the mean experimental adsorption capacity; and *N* is the total number of experimental observations. A better statistical fit was associated with higher *R*^2^ values and lower SSE and RMSE values. The kinetic models were used primarily as mathematical descriptions of the time-dependent adsorption behavior and were not considered, by themselves, conclusive evidence of a specific molecular adsorption mechanism.

### 2.5. Effect of pH

To analyze the effect of pH on adsorption, hydrochloric acid, acetic acid and sodium carbonate were used to adjust the pH of 1 g L^−1^ solutions to 4, 6 and 8 respectively at 311 K.

For each experiment, 1 mL of solution was placed into Eppendorf tubes, and the corresponding amount of MOF MIL-88D(Fe), previously determined in the adsorption kinetics study, was added.

### 2.6. Adsorption isotherms

Adsorption isotherms were used to describe the relationship between the amount of FA adsorbed on the surface of MIL-88D (Fe)-MOF and its equilibrium concentration in the solution at constant temperature and pressure.

To determine the optimal adsorption temperature, various FA solutions were prepared at concentrations ranging from 0.1 to 1 g L^−1^. Each solution was mixed with 30 mg of MIL-88D (Fe)-MOF and stirred for 100 min at 100 rpm. The temperatures used were 303, 311, and 318 K. The experimental results were then fitted to the Langmuir and Freundlich isotherm models.

The adsorption capacity was calculated from the mass balance:10
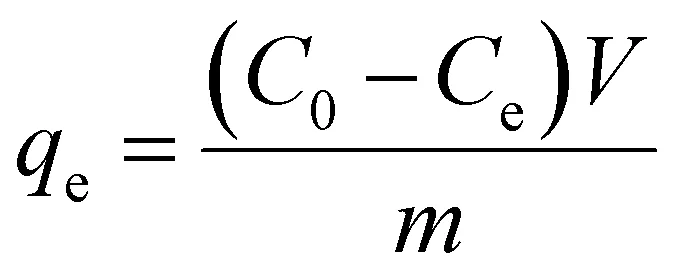
where *C*_0_ and *C*_e_ are the initial and residual FA concentrations, respectively (mg L^−1^), *V* is the solution volume (L), and *m* is the mass of MIL-88D(Fe) (g). The adsorption capacity, *q*_e_, is expressed in (mg g^−1^).

The experimental *q*_e_–*C*_e_ data obtained at each temperature were fitted directly to the nonlinear Langmuir and Freundlich isotherm models. The Langmuir equation was expressed as:11
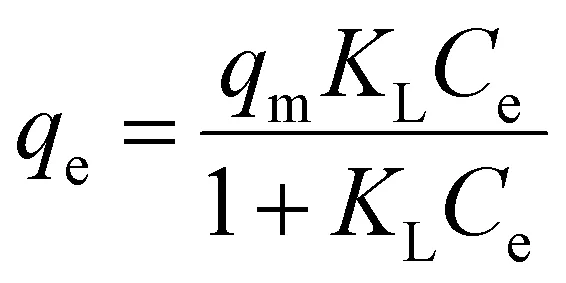
where *q*_m_ is the model-estimated saturation adsorption capacity (mg g^−1^) and *K*_L_ is the Langmuir affinity constant (L mg^−1^).

The Freundlich model was expressed as:

12 *q*_e_ = *K*_F_*C*_e_^1/*n*^

where *K*_F_ is the Freundlich adsorption constant and *n* is the Freundlich exponent describing the concentration dependence of adsorption.

The model parameters were estimated by nonlinear least-squares regression using the original, non-transformed *q*_e_ and *C*_e_ data. The quality of the fits was evaluated using the coefficient of determination (*R*^2^), the sum of squared errors (SSE), and the root-mean-square error (RMSE).

### 2.7. Adsorption of ferulic acid from nejayote

Nejayote, obtained from the “El Sol Naciente” (20°59′43.6″N 89°36′07.2″W) tortilla corn mill in the city of Mérida, was centrifuged at 5000 rpm for 10 min to remove solids, and the supernatant was recovered and stored at 253 K until use. The pH to be used for the removal of FA in the nejayote sample, as well as the amount of adsorbent and contact time, were obtained from the results obtained with the FA standard. The initial FA concentration in the nejayote was determined from triplicate analyses of the collected batch.

### 2.8. Ferulic acid quantification in nejayote samples

The concentration of ferulic acid in nejayote samples, before and after adsorption, was determined by high-performance liquid chromatography (HPLC). Samples were prepared by diluting one volume of nejayote with one volume of methanol (HPLC grade). The diluted samples were centrifuged at 3500 rpm to remove suspended solids, and the obtained supernatant was filtered using a 0.22 µm pore size filter. HPLC was performed with an Agilent 1100 system equipped with a G1377A/78A automatic injector, a G1376A capillary pump, and a G1315B ultraviolet detector. Separations were carried out using an Agilent C-18 column (3.5 µm, 4.6 × 100 mm). The mobile phases consisted of CH_3_CN/H_2_O (10 : 90) containing 5% (v/v) of HCOOH (phase A) and CH_3_CN/H_2_O (90 : 10) containing 5% of HCOOH (phase B). The gradient was 0 min 100% A, 15 min 90% A, 20 min 80% A, over 30 min at a flow rate of 1 mL min^−1^. UV absorbances of eluting peaks were recorded at 280 nm. For the desorption experiments, the FA-adsorbed MIL-88D(Fe) MOF was subjected to desorption by adjusting the pH of the solution with using 0.1 M NaOH solution (pH ≈ 11). The FA-loaded MIL-88D(Fe) was contacted with 5 mL of desorption solution for 60 min under stirring at 100 rpm. The desorption efficiency was calculated after centrifugation.

The reusability of MIL-88D(Fe) was evaluated through five consecutive adsorption–desorption cycles. Before the first adsorption cycle, the pH of the nejayote was adjusted from approximately 11.3 to 3.3 using 0.1 M HCl. Solutions of 0.1 M HCl and 0.1 M NaOH were used throughout the cycling experiments to adjust the pH during the adsorption and regeneration steps. For each adsorption cycle, 30 mg of MIL-88D(Fe) were placed in an Eppendorf tube and contacted with 1 mL of nejayote. The suspension was maintained under agitation at 38 °C (311 K) for 100 min. After the adsorption step, the sample was centrifuged at 5000 rpm for 5 min to separate the adsorbent from the liquid phase. The supernatant was collected and filtered through a 0.22 µm membrane prior to chromatographic analysis. For HPLC sample preparation, the recovered supernatant was mixed with 1 mL of HPLC-grade methanol before FA quantification. After each adsorption step, the recovered MIL-88D(Fe) was subjected to desorption in the same Eppendorf tube to minimize adsorbent losses during handling. For desorption, 1 mL of 0.1 M NaOH solution was added to the FA-loaded adsorbent, and the suspension was maintained under the same agitation and temperature conditions used during adsorption for 100 min. The alkaline medium was used to promote the release of adsorbed FA from the MIL-88D(Fe) surface. At the end of each desorption step, the adsorbent was separated by centrifugation and washed with distilled water to remove residual alkaline solution. The material was then reconditioned under acidic conditions using 0.1 M HCl before beginning the following adsorption cycle. FA removal efficiency for each adsorption cycle was calculated from the initial and residual FA concentrations determined by HPLC. All adsorption measurements were performed in triplicate. The initial FA concentration in the nejayote was also determined from three replicate measurements of the same collected batch.

## 3. Results and discussion

### 3.1. Characterization of MIL-88D (Fe)-MOF

The successful synthesis of the MIL-88D (Fe)-MOF was confirmed through XRD analysis. As shown in Fig. 1a, the diffraction pattern of the synthesized material exhibits characteristic peaks at 5.1°, 6.3°, 11°, and 12.8°, which are assigned to the (1̄00), (1̄11), (02̄1̄) and (02̄1̄) crystallographic planes, respectively. The excellent agreement between this pattern and the simulated (Fig. 1b) one reported in the literature^13,21^ confirms the formation of a pure, well-crystallized MIL-88D(Fe) narrow pore (np) phase.

**Fig. 1:**
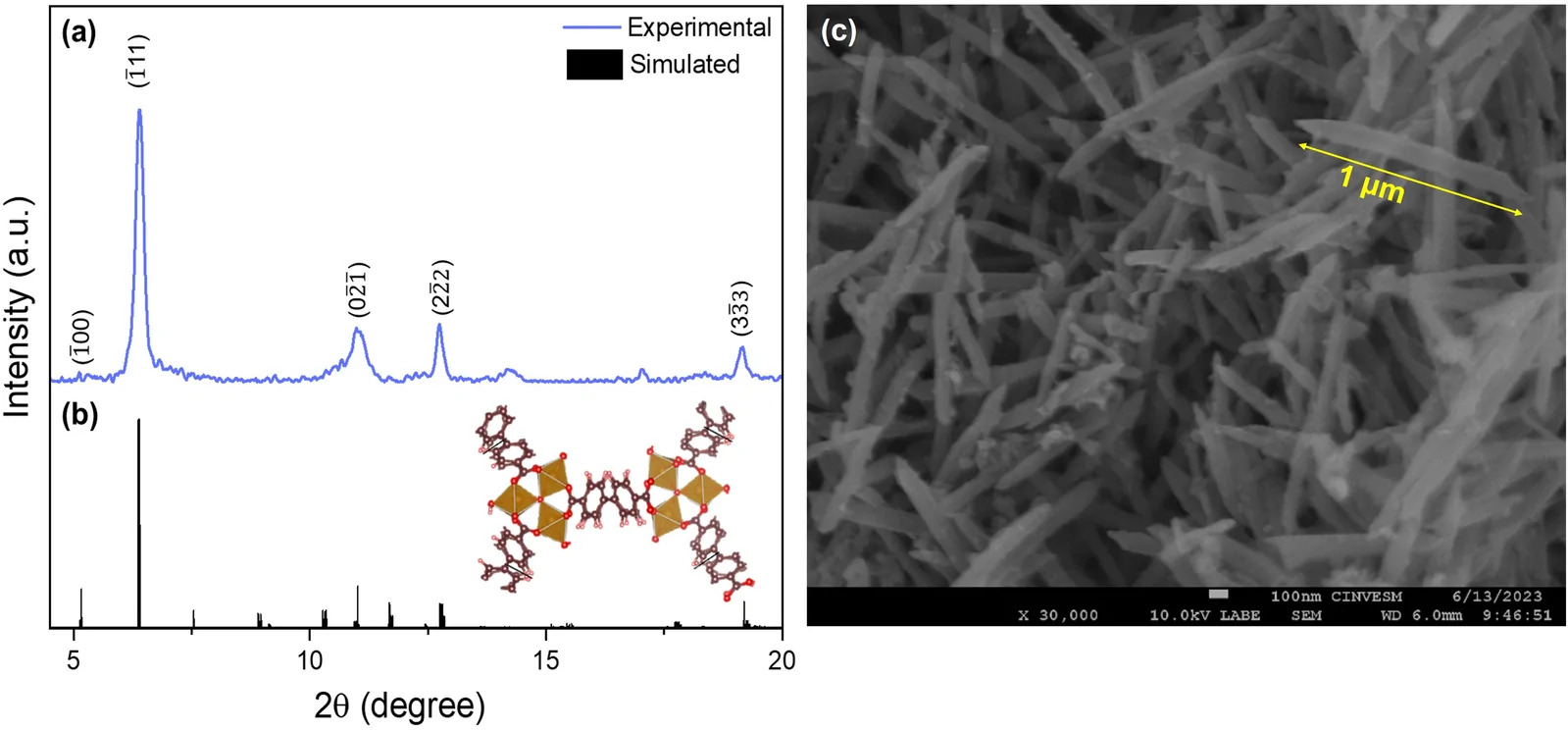
(a) XRD pattern as synthetized, (b) the simulated PXRD pattern for MIL-88D(Fe) np was generated from the computationally optimized structure reported by Horcajada *et al.*^22^ and used by Bara *et al.*^21^ and (c) SEM image of MIL-88D(Fe)-MOF.

MIL-88D(Fe) belongs to the flexible MIL-88 family, built from trinuclear iron(iii) oxo clusters coordinated to biphenyl-4,4′-dicarboxylate (BPDC) linkers.^21,23^ The sharp, well-defined peaks, especially at low angles, indicate a non-interpenetrated structure, enabling the framework's characteristic “breathing” behavior-reversible pore expansion and contraction.^21^ The high peak intensity and narrow FWHM reflect excellent crystallinity and long-range order, essential for consistent adsorption performance. The longer BPDC linkers increase pore size compared to other MIL-88 homologs (*e.g.*, fumarate or terephthalate), enhancing access for larger molecules like FA by providing larger pores and accessible active sites on iron-oxo clusters FA.^21^

SEM analysis (Fig. 1c) shows the MOF particles are primarily hexagonal rods (∼1 µm), with well-faceted surfaces and sharp edges, indicating high crystallinity consistent with XRD results. The morphology results from rapid microwave-assisted synthesis, which promotes uniform nucleation and growth, yielding discrete, non-agglomerated particles that are ideal for adsorption applications by maximizing surface accessibility.^21,24^ The lack of significant agglomeration and the presence of dispersed particles are advantageous for adsorption applications, as they ensure a high accessibility of the surface area and pore openings to the target analyte (FA).

The microwave-assisted solvothermal route employed in this work offers a significant reduction in synthesis time compared to conventional methods previously reported for MIL-88 MOFs. Conventional syntheses typically require between 12 and 24 hours of heating and frequently suffer from co-crystallization issues. For example, in the traditional solvothermal synthesis of MIL-88B, Ma *et al.* reported the formation of mixtures containing MIL-53 or MIL-101 impurities from the same starting solution, whereas their microwave-assisted synthesis provided superior-quality MIL-88B crystals with high yields and excellent phase purity.^25^ Similarly, Bara *et al.* reported the challenge in solvothermal synthesis of obtaining a mixture of non-interpenetrated MIL-88D(Fe) alongside the thermodynamic product, doubly interpenetrated MIL-126(Fe).^21^ In contrast, the present method yields pure-phase MIL-88D(Fe) in just 30 min. Beyond its thermal efficiency, microwave irradiation has been demonstrated to be a superior method for producing MOFs with smaller and more uniform particle sizes, higher phase purity, improved morphology, and greater yields.^26^ Despite this drastic reduction in reaction time, the obtained material exhibits crystallinity comparable to conventional reports, as confirmed by the XRD pattern (Fig. 1), and a morphology consistent with literature. A detailed comparison of the synthesis parameters and material properties is provided in Table 1. This demonstrates that microwave heating constitutes a highly efficient alternative for the rapid preparation of MIL-88D(Fe) without compromising its structural quality.

**Table 1:** Comparative synthesis of MIL-88 family solvothermal and microwave-assisted methods

Synthesized MOF	Synthesis method	Reaction time (min)	Reaction temperature (K)	Crystallinity and morphology	Yield (%)	Reference
MIL-88A(Fe)	Solvothermal	1440	373	Micrometric elongated rod-like crystals of around 50 × 300 µm	83	27
MIL-88A(Fe)	Microwave	5	353	Elongated rod-like nanocrystals of around 100 × 30 nm	61	27
MIL-88B(Fe)	Solvothermal	720	383	Regular hexagonal diamond-like structure with a diameter of approximately 500 nm	78	28
MIL-88B(Fe)	Microwave	10	423	Spindle-shaped crystals with high dispersibility and a uniform size of *ca.* 2.4 µm in length and 1.2 µm in diameter	35	25
MIL-88D(Fe)	Solvothermal	1440	393	Spindle-like structure, 13 µm in length	82	29
MIL-88D(Fe)	Microwave	30	373	Hexagonal rods 1 µm in length	52	This work

The N_2_ adsorption isotherm (Fig. 2) shows a Type II pattern,^30^ typical of non-porous or macroporous materials. The BET surface area is about 68.5 m^2^ g^−1^,^31^ which is low but expected for this MOF. The low surface area results from the framework's ‘breathing’ behavior: after synthesis, the structure is in a contracted np form,^21,32^ caused by flexible hinges at the Fe trimers and linkers. This flexibility leads to pore collapse, reducing N_2_ uptake. The value aligns with literature for the closed form of MIL-88D(Fe),^14,21^ confirming the framework's nature and ruling out a rigid, permanently open structure.

**Fig. 2:**
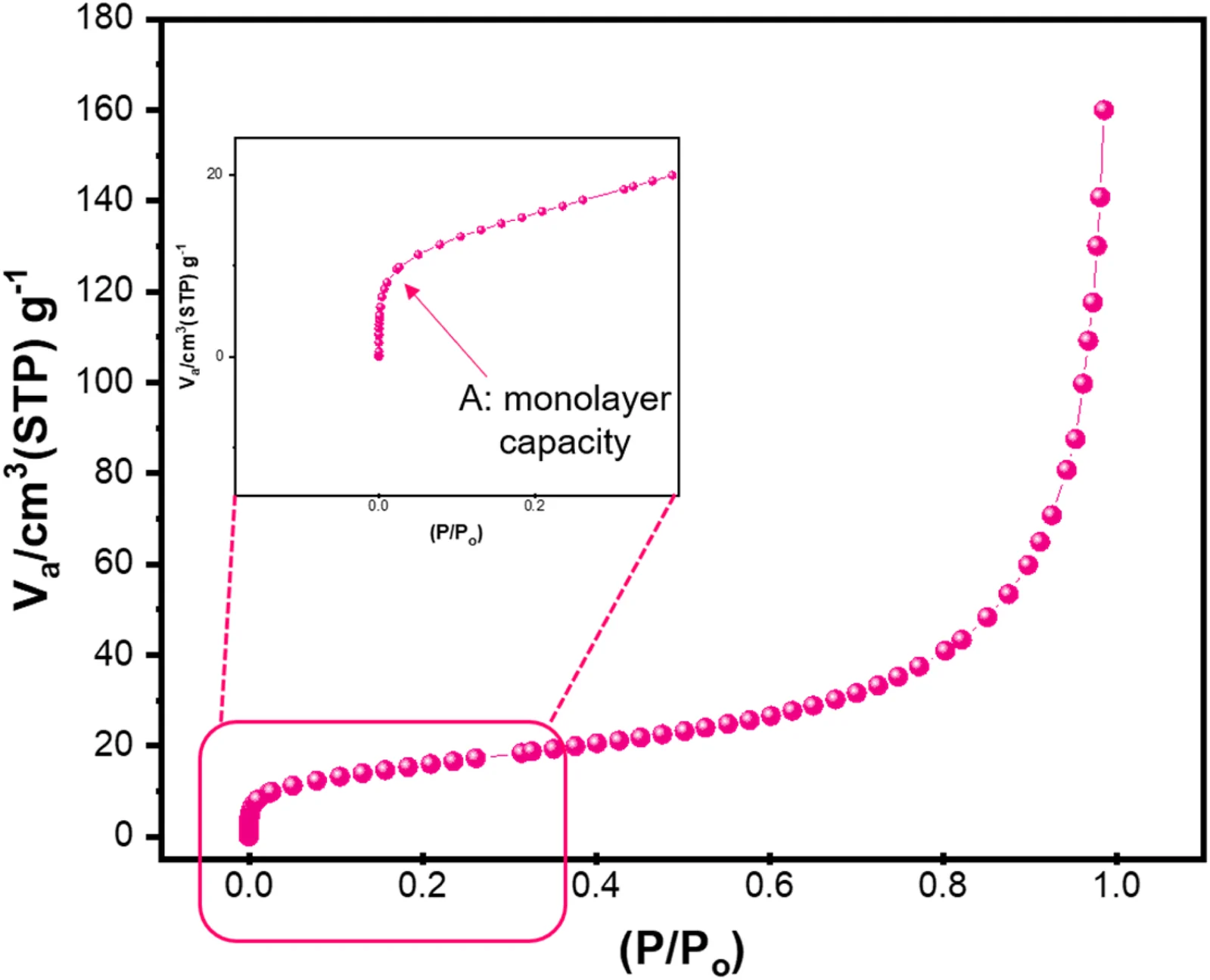
N_2_ adsorption isotherm of the MIL-88D (Fe) np.

### 3.2. Adsorption kinetics of ferulic acid on MIL-88D (Fe)-MOF

The adsorption kinetics of FA onto the MIL-88D(Fe) surface were investigated by monitoring the solute concentration over time using varying adsorbent doses (Fig. 3a). As anticipated, an increase in the MOF mass (from 5 to 30 mg) resulted in a higher removal efficiency, which is attributed to the greater availability of adsorption sites. Notably, the time required to reach equilibrium remained consistent at approximately 60 min across all tested doses, indicating that the adsorption rate is not limited by mass transfer effects under these conditions.

**Fig. 3:**
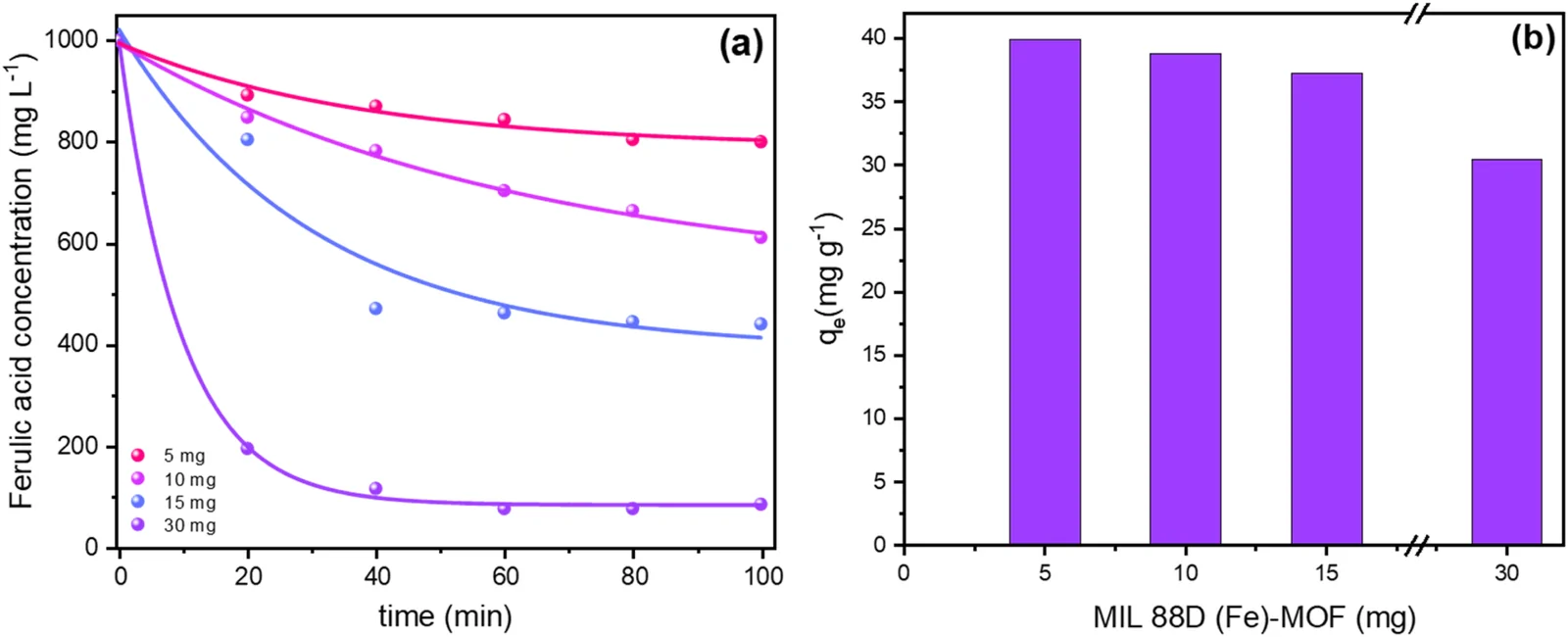
(a) Adsorption kinetics of FA from different amounts and (b) equilibrium adsorption capacity (*q*_e_) of FA at different doses of MIL-88D(Fe)-MOF.

The relationship between adsorbent dose and the equilibrium adsorption capacity (*q*_e_) is shown in Fig. 3b. The results indicate that the adsorption capacity decreases as the adsorbent dose increases, which is a typical phenomenon attributed to site overcrowding or unsaturated adsorption sites at higher doses. Despite this decrease in capacity, the total removal efficiency increased with dose, reaching a maximum of 91.53% with 30 mg of MIL-88D(Fe), demonstrating the material's high effectiveness for FA sequestration under the specified conditions.

The experimental kinetic data were subsequently fitted directly to the nonlinear pseudo-first-order (PFO), pseudo-second-order (PSO), and Elovich equations described in Section 2.4. Direct nonlinear regression was used instead of fitting transformed linear expressions because linearization can alter the experimental error structure and influence the estimated kinetic parameters. Recent critical analyses of adsorption kinetic modeling recommend nonlinear fitting and the use of multiple statistical criteria rather than relying exclusively on correlation coefficients obtained from linearized equations.^18–20^

The corresponding nonlinear fits are shown in Fig. 4, and the fitted parameters are summarized in Table 2.

**Fig. 4:**
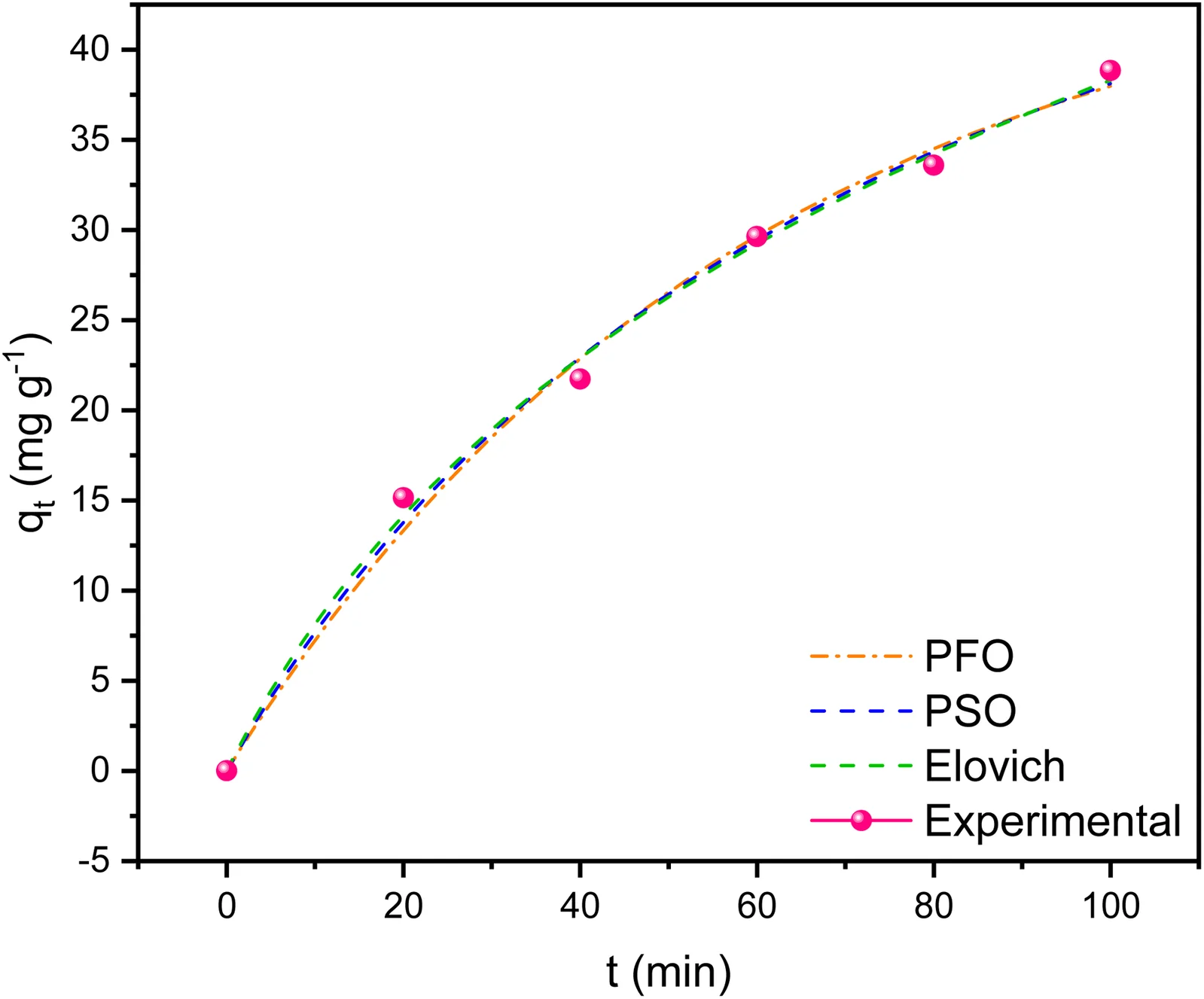
Experimental adsorption kinetics of FA on MIL-88D(Fe) and nonlinear fits of the pseudo-first-order (PFO), pseudo-second-order (PSO), and Elovich kinetic models. Symbols represent experimental data and curves represent nonlinear model predictions.

**Table 2:** Kinetic model parameters and goodness-of-fit statistics for FA adsorption on MIL-88D(Fe)

Model	Fitted parameters	*R* ^2^	RMSE (mg g^−1^)	SSE (mg g^−1^)^2^
PFO	*q*_e_ = 46.61 mg g^−1^	0.9938	1.014	6.170
	*k*_1_ = 0.01685 min^−1^			
PSO	*q*_e_ = 68.31 mg g^−1^	0.9956	0.859	4.431
	*k*_1_ = 1.848 × 10^−4^ g mg^−1^ min^−1^			
Elovich	*α* = 0.974 mg g^−1^ min^−1^	0.9968	0.728	3.182
	*β* = 4.30 × 10^−2^g mg^−1^ min^−1^			
Weber–Morris	*k*_id_ = 3.884 mg g^−1^ min^−1/2^	0.9929	1.091	7.137
	*C* = −1.107 mg g^−1^			

These results indicate that all three models describe the experimental kinetic profile reasonably well, although the Elovich model gave the best statistical performance according to the combined *R*^2^, RMSE, and SSE criteria. The differences between the Elovich and PSO fits are nevertheless relatively small, and therefore the kinetic behavior should not be assigned exclusively to a single model. It is also important to note that the model-derived equilibrium capacities obtained from PFO and PSO, *q*_e_ = 46.61 and *q*_e_ = 68.31 mg g^−1^, respectively are higher than the highest experimentally measured value of *q*_e_ = 68.31 mg g^−1^are higher than the highest experimentally measured value of *q*_*t*_ = 38.85 mg g^−1^ at 100 min. This difference reflects extrapolation of the fitted kinetic functions beyond the experimental contact period, particularly because a well-defined plateau was not reached. Therefore, these *q*_e_ values should be regarded as model-estimated parameters rather than experimentally demonstrated equilibrium capacities.

The slightly better statistical performance of the Elovich model is consistent with a kinetic profile in which the adsorption rate progressively changes as site occupancy increases and adsorption environments with different interaction energies may contribute to uptake. However, neither the Elovich nor the PSO fit alone provides sufficient evidence to establish a specific molecular adsorption mechanism. Although PSO has historically been associated with chemisorption in adsorption literature, more recent analyses have shown that PFO and PSO equations are empirical or semi-empirical descriptions that may successfully reproduce adsorption kinetics governed by different combinations of surface reaction and mass-transfer phenomena.^20^

The possible contribution of intraparticle mass transfer was further examined using the Weber–Morris relationship (Fig. 5 and Table 2). Although the general Weber–Morris relationship provides a good empirical description of the available data, the model must be interpreted with caution because a linear relationship between *q*_*t*_ and *t*^1/2^ does not, in itself, establish intraparticle diffusion as the sole rate-controlling mechanism.^32,33^ Wu *et al.* and, more recently, Wang and Guo have specifically discussed the limitations associated with using the IPD model to assign individual mass transfer mechanisms.^33,34^

**Fig. 5:**
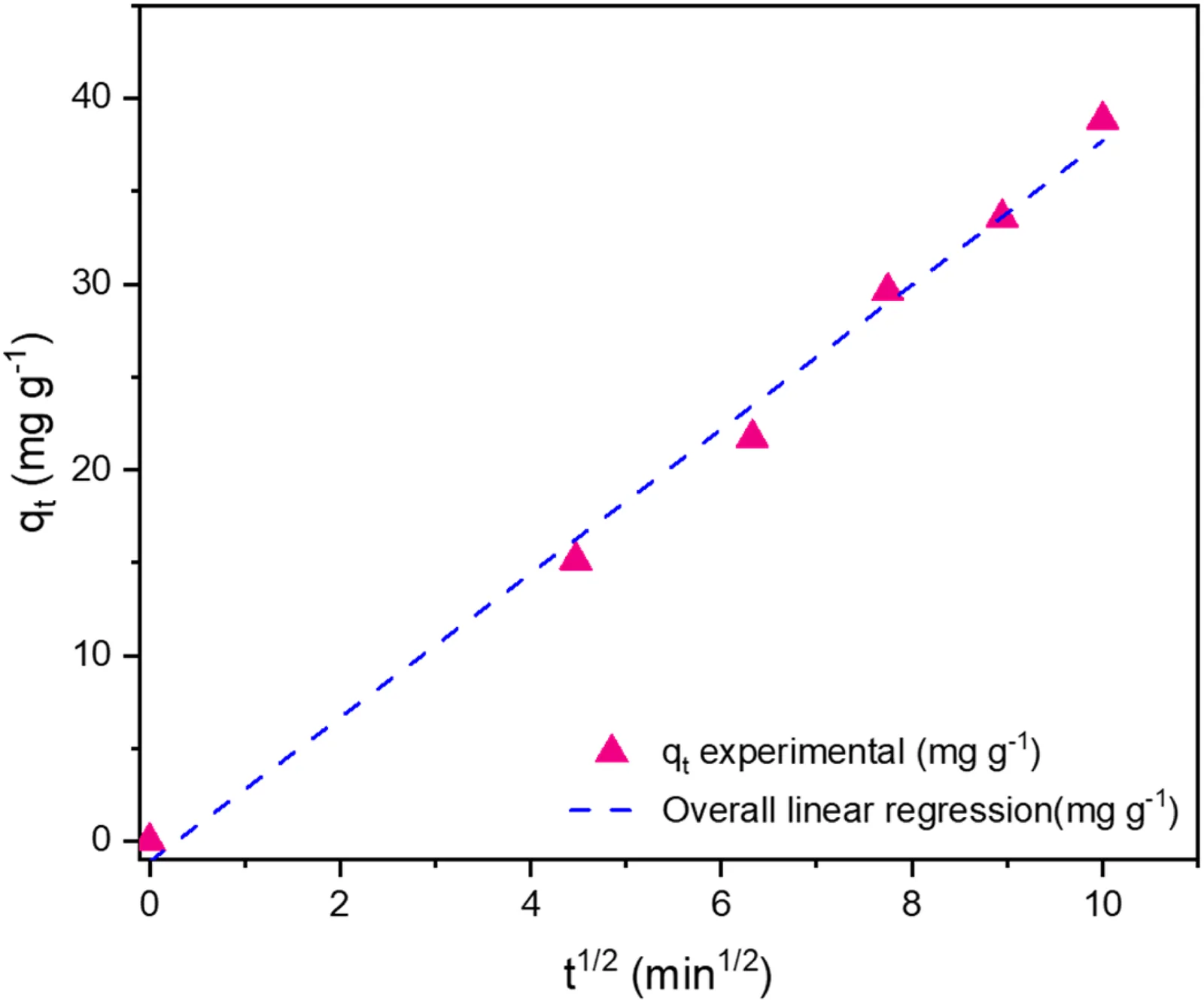
Weber–Morris intraparticle diffusion analysis for FA adsorption on MIL-88D(Fe). Symbols represent experimental data and the solid line represents the overall linear regression of *q*_*t*_*versus t*^1/2^. Because of the limited number of experimental time points, individual diffusion regions were not independently fitted.

Overall, the nonlinear kinetic analysis indicates rapid initial FA uptake followed by a progressively slower adsorption rate over the 100 min contact period. The Elovich equation provided the lowest statistical fitting error, closely followed by PSO, whereas the Weber–Morris analysis suggests that mass-transfer phenomena may contribute to the observed adsorption kinetics. However, the present kinetic data does not support assigning either chemisorption or intraparticle diffusion as the only rate-controlling mechanism.

### 3.3. Effect of pH on adsorption

The adsorption capacity of MIL-88D(Fe) for FA was highly dependent on pH (Fig. 6). The maximum removal efficiency of 91.62% (corresponding to an uptake of 30.54 mg g^−1^) was achieved at pH 3.3. In order to evaluate the system's behavior, the surface charge of MIL-88D(Fe) was determined *via* zeta potential measurements across a pH range of 2 to 10 (Fig. 6b). The point of zero charge (pH_PZC_) was found to be approximately 7. At pH 3.3, the positive zeta potential is strongly positive (+25 mV) demonstrating that the MOF surface is positively charged under the optimum adsorption conditions. In paralell, FA (p*K*_a_ ≈ 4.5) exists predominantly in its protonated, neutral form under these conditions. Therefore, electrostatic attraction is not expected to be the primary driving force for adsorption at pH 3.3. Instead, the adsorption process is more consistent with specific host–guest interactions, particularly π–π interactions between the aromatic ring of ferulic acid and the BPDC linker, together with hydrogen bonding involving the hydroxyl and carboxylic groups of ferulic acid and oxygen-containing sites of the framework, as further supported by the FTIR results discussed below.

**Fig. 6:**
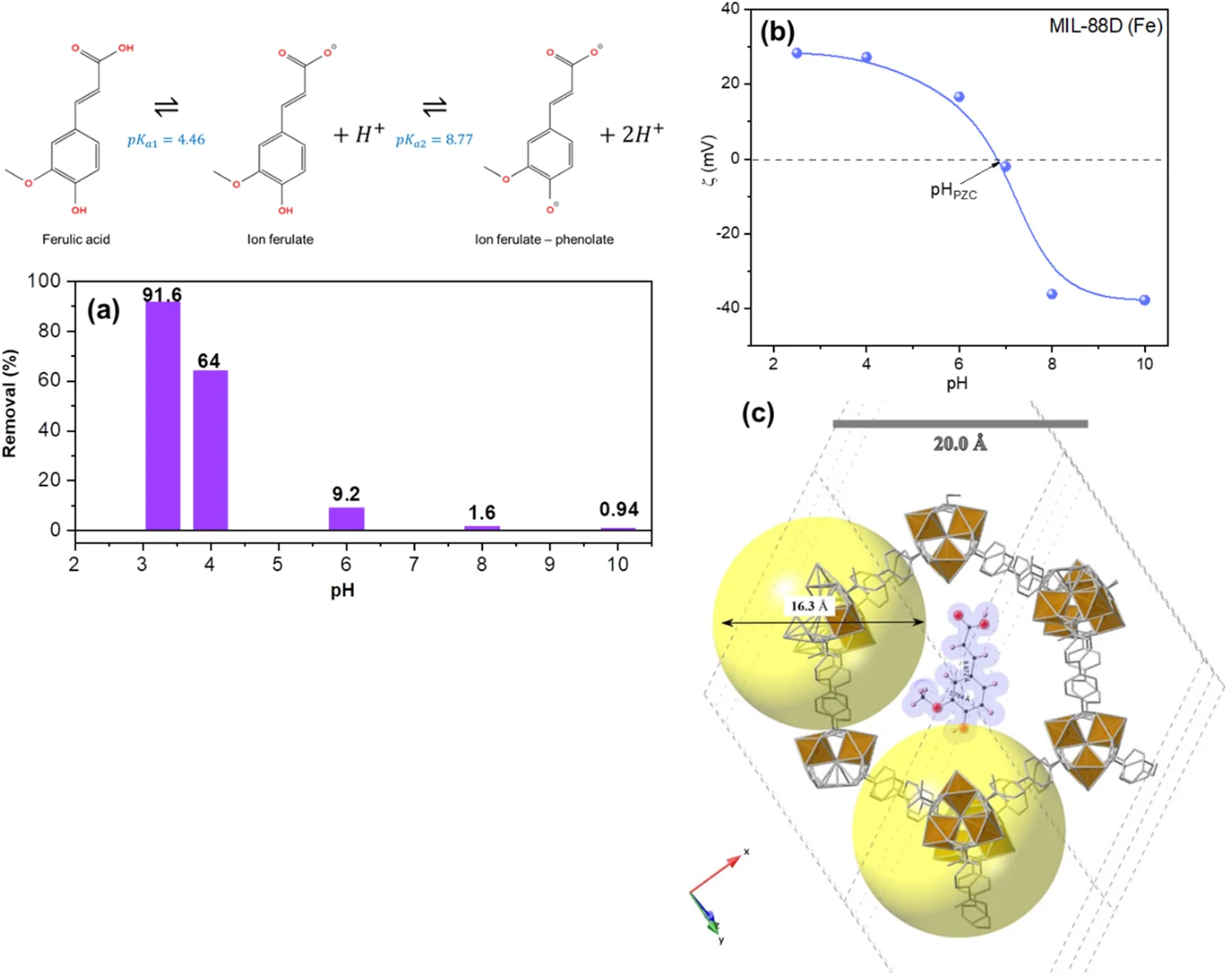
(a) Effect of pH on FA removal by MIL-88D (Fe)-MOF. The insert in the upper part of the plot illustrates the chemical equilibrium of FA as a function of the pH. (b) Zeta potential of MIL-88D(Fe)-MOF as a function of pH. (c) Crystal structure of the Fe-based MOF MIL-88B viewed along the [110] direction, showing the main porous channels with their dimensions drawn to scale (Å) and a ferulic acid molecule adsorbed within the pore. Orange polyhedrons represent the FeO_6_ octahedra of the trimeric metal clusters, bridged by 1,4-benzenedicarboxylate linkers; carbon atoms are shown in grey, oxygen in red and hydrogen in white. Structural models were built from the CIF files of each system reported in ref. 22 and 35 and the representation was rendered with Crystal Maker® software v11.7.4 (Personal Educational License).

A sharp decrease in adsorption capacity was observed as the pH increased from 4 to 10, with the uptake declining to merely 0.313 mg g^−1^ at alkaline pH. This behavior correlates directly with the pH-dependent speciation of both the adsorbate and the adsorbent. Above the p*K*_a_ of ferulic acid, the molecule progressively converts into the negatively charged ferulate anion. Simultaneously, as the pH approaches and exceeds the pHPZC, the zeta potential measurements show that the MIL-88D(Fe) surface loses its positive character and becomes negatively charged. Under these conditions, electrostatic repulsion between ferulate anions and the negatively charged MOF surface becomes increasingly unfavorable for adsorption. The deprotonation of surface and adsorbate functional groups may additionally reduce the strength of hydrogen-bonding interactions, further contributing to the marked decrease in uptake. Collectively, these results demonstrate that FA adsorption on MIL-88D(Fe) is strongly governed by pH-dependent adsorbate speciation and surface-charge effects, with electrostatic repulsion becoming a major limiting factor at alkaline pH. The adsorption mechanism, driven by hydrogen bonding and π–π interactions, is summarized schematically in Fig. 6c.^36,37^

### 3.4. Nonlinear adsorption isotherms and temperature-dependent adsorption behavior of ferulic acid

The adsorption behavior of ferulic acid (FA) on MIL-88D(Fe) was evaluated at 303, 311, and 318 K using the experimental *q*_e_–*C*_e_ data obtained after the 100 min contact period. The corresponding nonlinear fits are shown in Fig. 7, and the fitted parameters are summarized in Table 3.

**Fig. 7:**
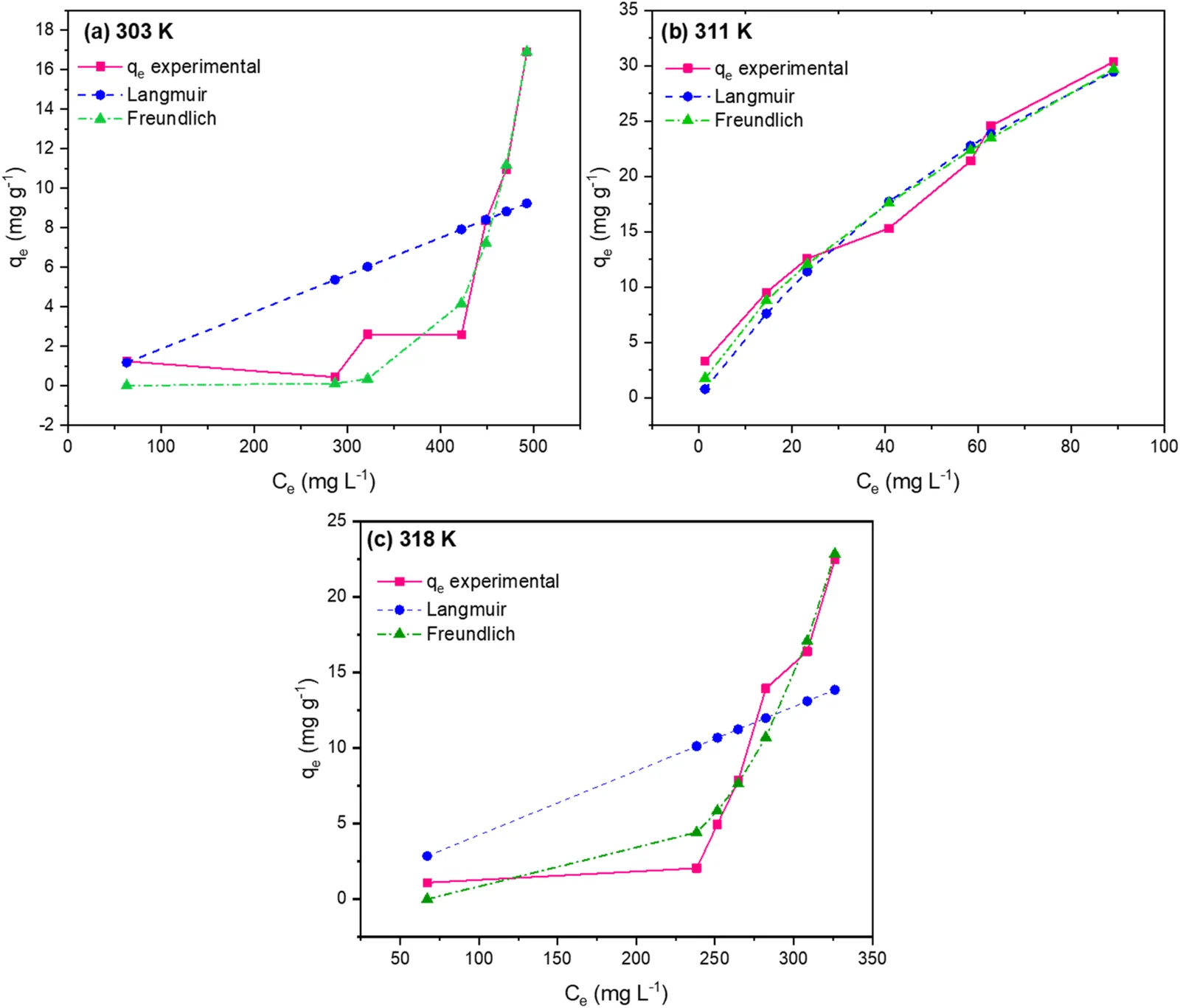
Nonlinear adsorption isotherms of ferulic acid on MIL-88D(Fe) at (a) 303 K, (b) 311 K, and (c) 318 K. Symbols represent experimental *q*_e_–*C*_e_ data, whereas the continuous curves represent the nonlinear Freundlich and Langmuir model fits.

**Table 3:** Nonlinear Langmuir and Freundlich parameters for FA adsorption on MIL-88D(Fe) at different temperatures^a^

*T* (K)	Model	Fitted parameters	*R* ^2^	RMSE (mg g^−1^)	SSE (mg g^−1^)^2^
303	Langmuir	*q*_m_ = N.R.	0.4333	4.274	127.87
		*K*_L_ = N.R.			
303	Freundlich	*n* = 0.110	0.9529	1.232	10.62
		*K*_F_ = 4.99 × 10^−24^			
311	Langmuir	*q*_m_ = 66.84 mg g^−1^	0.9607	1.710	20.48
		*K*_L_ = 0.00884 L mg^−1^			
311	Freundlich	*n* = 1.491	0.9790	1.251	10.96
		*K*_F_ = 1.461			
318	Langmuir	*q*_m_ = N.R.	0.4757	5.361	201.21
		*K*_L_ = N.R.			
318	Freundlich	*n* = 0.191	0.9515	1.631	18.61
		*K*_F_ = 1.56 × 10^−12^			

a N.R.: not reliably determined. The nonlinear Langmuir regression did not provide physically identifiable *q*_m_ and *K*_L_ estimates because the experimental data did not exhibit a sufficiently defined saturation region.

A clear temperature dependence was observed, although the response was not monotonic. Among the investigated temperatures, the highest experimentally measured adsorption capacity was obtained at 311 K, reaching *q*_e_ = 30.36 mg g^−1^ at *C*_e_ = 89.11 mg L^−1^. Lower adsorption capacities were observed at both 303 K and 318 K. Consequently, 311 K represents the optimal experimental temperature within the investigated range, rather than indicating whether the adsorption process is simply continuously endothermic or exothermic.

The experimental data at each temperature were fitted directly to the nonlinear Langmuir and Freundlich equations described in Section 2.6. Direct fitting of the original *q*_e_–*C*_e_ data was preferred over linearized representations because mathematical transformation can modify the experimental error structure and influence parameter estimation. Nonlinear regression, together with complementary goodness-of-fit criteria, is widely recommended for adsorption-isotherm analysis.^30,38^

As shown in Table 3, at 311 K, the Freundlich model provided the best statistical description of the experimental data based on the combined criteria of *R*^2^, RMSE, and SSE. The value *n* = 1.491 > 1 is consistent with favorable adsorption behavior over the investigated concentration range. However, the better Freundlich fit should not be interpreted as direct proof of multilayer adsorption. Rather, it indicates that a heterogeneous distribution of adsorption environments or interaction energies provides a more appropriate empirical description than the ideal homogeneous-site assumptions associated with the Langmuir model.^30^

An important distinction must also be made between the highest experimentally measured adsorption capacity and the Langmuir-derived saturation parameter. The value 30.36 mg g^−1^ is the highest adsorption capacity measured at 311 K, whereas *q*_m_ = 66.84 mg g^−1^ is obtained by extrapolation of the nonlinear Langmuir equation. Because a clear saturation plateau was not experimentally reached, this *q*_m_ value should be considered a model-estimated saturation parameter rather than an experimentally demonstrated maximum adsorption capacity. In addition, at 303 and 318 K, the Freundlich model provided a considerably better statistical description than Langmuir model.

Importantly, the nonlinear Langmuir regressions at 303 and 318 K did not provide physically identifiable estimates of *q*_m_ and *K*_L_. In both cases, the optimization tended toward very large *q*_m_ values accompanied by extremely small *K*_L_ values. This behavior results from the absence of sufficient curvature and an experimentally defined saturation plateau, meaning that multiple combinations of *q*_m_ and *K*_L_ can describe nearly the same portion of the experimental curve. Consequently, the corresponding Langmuir parameters at 303 and 318 K were not considered physically meaningful and were not used for mechanistic or thermodynamic interpretation.

Although the Freundlich model provided a better empirical fit at 303 and 318 K, the corresponding values of *n* = 0.110 and *n* = 0.191, respectively, are significantly lower than unity, indicating an adsorption response that is strongly concentration-dependent and exhibits upward curvature. Consequently, these parameters should be interpreted primarily as empirical descriptors of the measured isotherms rather than as evidence of a specific adsorption mechanism. More generally, adsorption isotherm models should be used with caution when experimental data do not fully satisfy the corresponding assumptions.^30^

The non-monotonic temperature response should also not be interpreted as evidence that specific FA-MIL-88D(Fe) bonds are broken between 311 and 318 K. MIL-88 materials belong to a well-established family of structurally flexible MOFs whose unit-cell dimensions and accessible pore environments can respond strongly to guest molecules and solvent uptake. MIL-88 frameworks exhibit guest-dependent swelling while retaining the underlying coordination framework. Flexible MOFs can also respond to temperature, guest adsorption, solvent evacuation, and other external stimuli through structural rearrangements without requiring cleavage of the coordination network.^32,39,40^

Importantly, large structural responses in the MIL-88 family do not necessarily require breaking the coordination framework. For MIL-88-type structures, reversible pore expansion and contraction can involve large atomic displacements while preserving the framework topology, and these transformations are governed by a balance between host–guest interactions and intrinsic framework flexibility. Consequently, a relatively small temperature change can modify or alter the relative stability and the number of accessible configurations of the structure, the solvation of guest species, and the accessibility of adsorption sites, without entailing the thermal rupture of a strong bond between the adsorbate and the adsorbent.^41^

Temperature-dependent, non-monotonic adsorption has also been experimentally demonstrated in other flexible MOFs. Wang *et al.*, described a flexible structure that exhibited a characteristic adsorption maximum upon reaching a specific temperature threshold; “*in situ*” diffraction revealed that this unusual behavior was due to structural “breathing”, dependent on both guest molecules and temperature, rather than simple bond breaking. Therefore, although the current MIL-88D(Fe)/FA system is chemically different, the literature demonstrates that adsorption maxima within relatively narrow temperature ranges are physically reasonable for flexible structures.^42^

In this system, the highest experimentally measured FA uptake was observed at 311 K, whereas lower values were obtained at both 303 and 318 K. Consequently, we interpret 311 K as the experimental optimum within the studied temperature range, rather than as evidence of a specific bond-strength threshold. The observed behavior could reflect a combination of factors: structural flexibility, and various possible host–guest interactions, including hydrogen bonding, π–π interactions. Since no temperature-resolved “*in situ*” structural or spectroscopic measurements were performed in this study, the origin of this non-monotonic response cannot be attributed exclusively to a single interaction or structural transition.

### 3.5. Adsorption of ferulic acid from nejayote

The practical applicability of MIL-88D(Fe) was evaluated for the recovery of FA from nejayote samples obtained from a local corn mill. During nixtamalization, the alkaline conditions partially solubilize hemicellulose, releasing bound FA into the wastewater. The initial FA concentration in the nejayote, determined by HPLC from triplicate analyses of a single collected batch, was 0.497 ± 0.012 g L^−1^, with a total phenolic content of 0.551 g L^−1^, indicating that FA constitutes approximately 90% of the phenolic compounds present in this effluent stream (Fig. 8a). It should be noted that as a real agro-industrial effluent, the FA concentration in nejayote is subject to batch-to-batch variations inherent to the nixtamalization process. Therefore, the reported uncertainty reflects the analytical repeatability under our experimental conditions rather than the temporal variability across different production days.

**Fig. 8:**
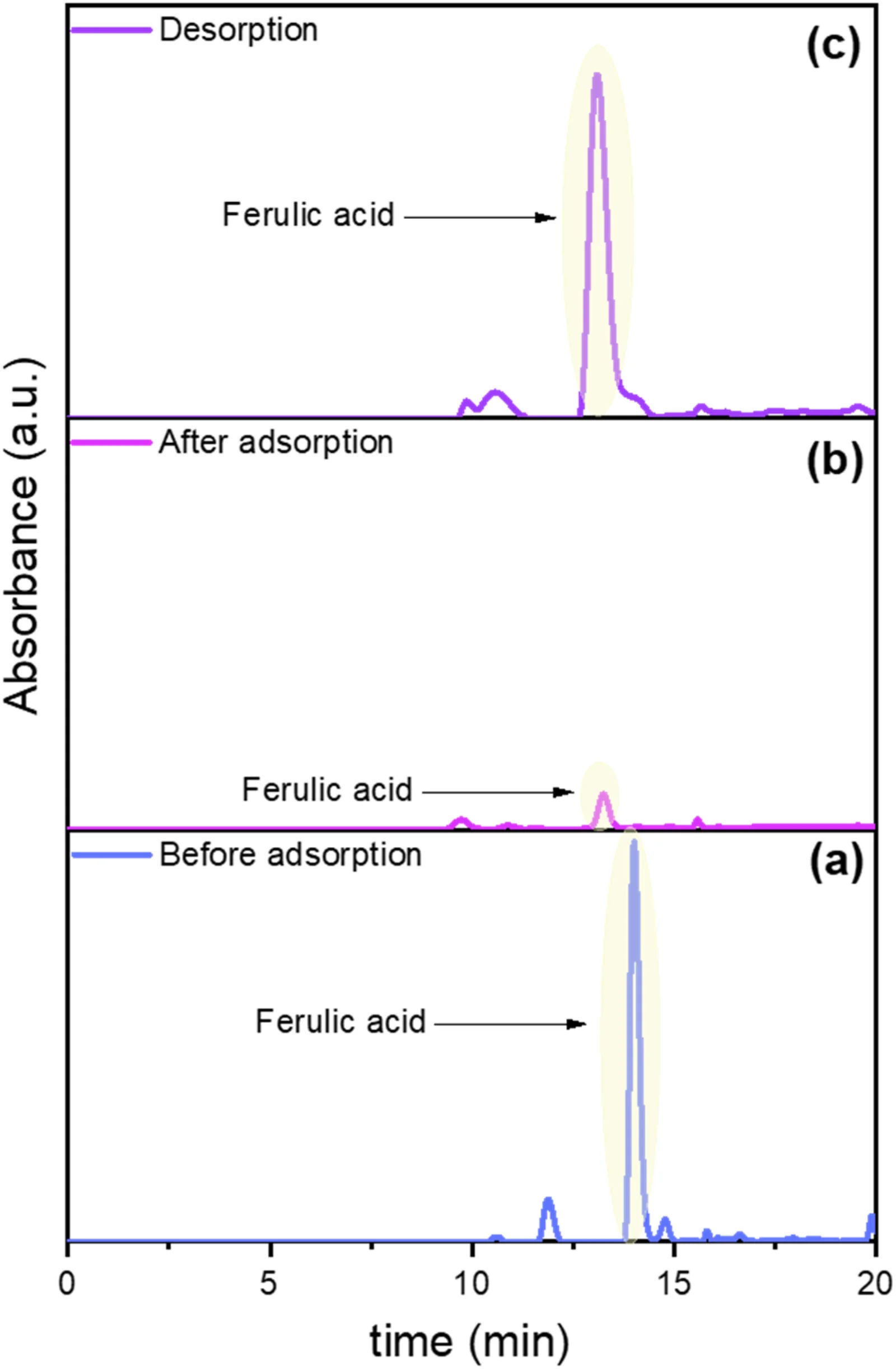
Chromatogram of FA: (a) before and (b) after applying optimal adsorption parameter, (c) desorption. Different adsorbents have been employed for the recovery of FA and other phenolic compounds 4 (Table 4). MIL-88D(Fe) exhibits markedly faster adsorption kinetics, reaching equilibrium within 60 min. This is significantly shorter than the times required by conventional adsorbents such as mesoporous carbon (90 min),^43^ Amberlite XAD-4 (90 min),^44^ and other MOFs like MFM-300(Sc),^45^ which typically require between 90 min to several days to reach equilibrium.^46^ This rapid uptake is a distinct advantage for practical, large-scale applications where processing time is a critical economic factor.

The adsorption experiment was conducted under optimized conditions established from standard solutions. Under these conditions, the FA concentration decreased from an initial value of 0.4967 g L^−1^ to 0.0480 g L^−1^ after adsorption, corresponding to an adsorption efficiency of 90.34% (Fig. 8b), with an adsorption capacity of 14.96 mg g^−1^. Following the adsorption step, the FA-adsorbed MIL-88D(Fe) MOF was subjected to desorption by adjusting the pH to 11 with concentrated sodium hydroxide. The concentration of FA after desorption was 0.3685 g L^−1^ (Fig. 8c).

The overall recovery of FA, relative to the initial concentration in the nejayote, was approximately 74.19%. This demonstrates that MIL-88D(Fe) effectively adsorbs and releases FA from complex wastewater matrices, highlighting its potential for phenolic compound recovery in industrial effluent treatment processes (Table 4).

**Table 4:** Overview of adsorbents for ferulic acid

Adsorbent	Adsorption capacity (mg g^−1^)	Adsorption isotherm	Kinetics	Selectivity	Regeneration mechanism	Reference
MIL-140C (Zr-MOF)	231.48	Langmuir	Pseudo-second order	Phenolic compounds and furfural even in the presence of excessive amounts of sugars such as xylose and glucose	Ethanol for desorption, excellent reusability over 5 consecutive adsorption/desorption cycles	47
Mesoporous carbon	238.5	Freundlich	Pseudo-first order	Non-selective to ferulic acid in the presence of other phenolic acids	Desorption using a mixed solvent (NaOH/ethanol), and reusability is not detailed	43
ZIF-8-HCP (hypercrosslinked composite)	15.58	Langmuir	Pseudo-second order	Affinity towards phenolic acids in complex vegetal matrices	Triethylamine for desorption and reusability is not detailed	48
Amberlite XAD-16 (styrenic resin)	133	Freundlich	Crank	Affinity towards non-ionized compounds	—	44
M-1 (core–shell magnetic lignin)	6.67	Freundlich	Pseudo-second order	Selectivity for ferulic acid and furfural over monosaccharides (glucose, xylose, arabinose), acetic acid, and sulfuric acid	Desorption using NaOH, and it has excellent reusability over four cycles	49
MIL-88D (Fe-MOF)	30.36	Freundlich	Pseudo-second order	Selectivity towards phenolic acids over starches and hemicellulose	Desorption using NaOH, and it has excellent reusability over five cycles	This work

Furthermore, the high removal efficiency achieved in real nejayote (90.3%) demonstrates the remarkable robustness of MIL-88D(Fe). It effectively absorbs FA even in the presence of high organic and salt content characteristic of this complex wastewater. This performance indicates that competitive adsorption and matrix effects do not significantly hinder its efficacy, a common limitation for many conventional adsorbents.

Although the maximum adsorption capacity of MIL-88D(Fe)-MOF for standard solutions (30.36 mg g^−1^) is lower than some values reported for other high-porosity materials, including certain MOFs, this can be rationalized by the nature of host–guest interactions. We hypothesize that the framework engages in moderate-strength interactions (*e.g.*, coordination with open metal sites, π–π stacking and hydrogen bonding interactions) with FA. In contrast, systems reporting exceptionally high capacities often rely on strong, sometimes irreversible, which can lead to rapid saturation and challenges in adsorbent regeneration and solute recovery.

The low BET surface area (68.5 m^2^ g^−1^) measured by N_2_ adsorption at 77 K is characteristic of the closed-pore (cp) form of MIL-88D(Fe), where the framework remains kinetically trapped in a contracted state at cryogenic temperature. However, under our adsorption conditions (308–311 K, aqueous phase), three synergistic factors enable pore opening and FA access: (i) the higher temperature provides sufficient thermal energy to overcome the activation barrier for framework expansion; (ii) FA molecules establish attractive host–guest interactions (π–π stacking, hydrogen bonding, and electrostatic interactions) that stabilize the open-pore form, unlike inert N_2_ which cannot engage in such specific interactions; and (iii) the aqueous solvent facilitates framework swelling through solvent–framework interactions.^21,50^

Fig. 9 shows that the MIL-88D(Fe) loaded with FA provides further insights into the structural response of the framework. It illustrates a classic and fascinating breathing effect,^21^ characteristic of the flexible MIL-88 family.^21^ Specifically, the initial peaks observed above ∼6.5°, and at ∼11° and ∼12.5° (2*θ*), corresponding to the low-density planes of the as-prepared, expansive (large pore – lp) structure, diminish or nearly disappear after FA adsorption (Fig. 9a).^50,51^ This indicates a loss of crystalline periodicity at these interplanar distances due to solvent removal or molecular reordering. Conversely, new sharp peaks emerge at around 17°, 25.5°, and 27° (2*θ*), signifying a phase transition into a collapsed or np state (Fig. 9b).^52^ The emergence of the peak at ∼17° suggests homogeneous contraction of the unit cells, compacting the metal skeleton, while the increased intensity of peaks near 25°–27° reflects successful adsorption, involving ordered packing and π–π interactions between aromatic rings of the MOF ligands and FA. Importantly, the persistence of sharp, well-defined peaks confirms that this transition is reversible; the framework remains crystalline and intact, albeit in a different phase, accommodating the guest molecules within its channels.^26^

**Fig. 9:**
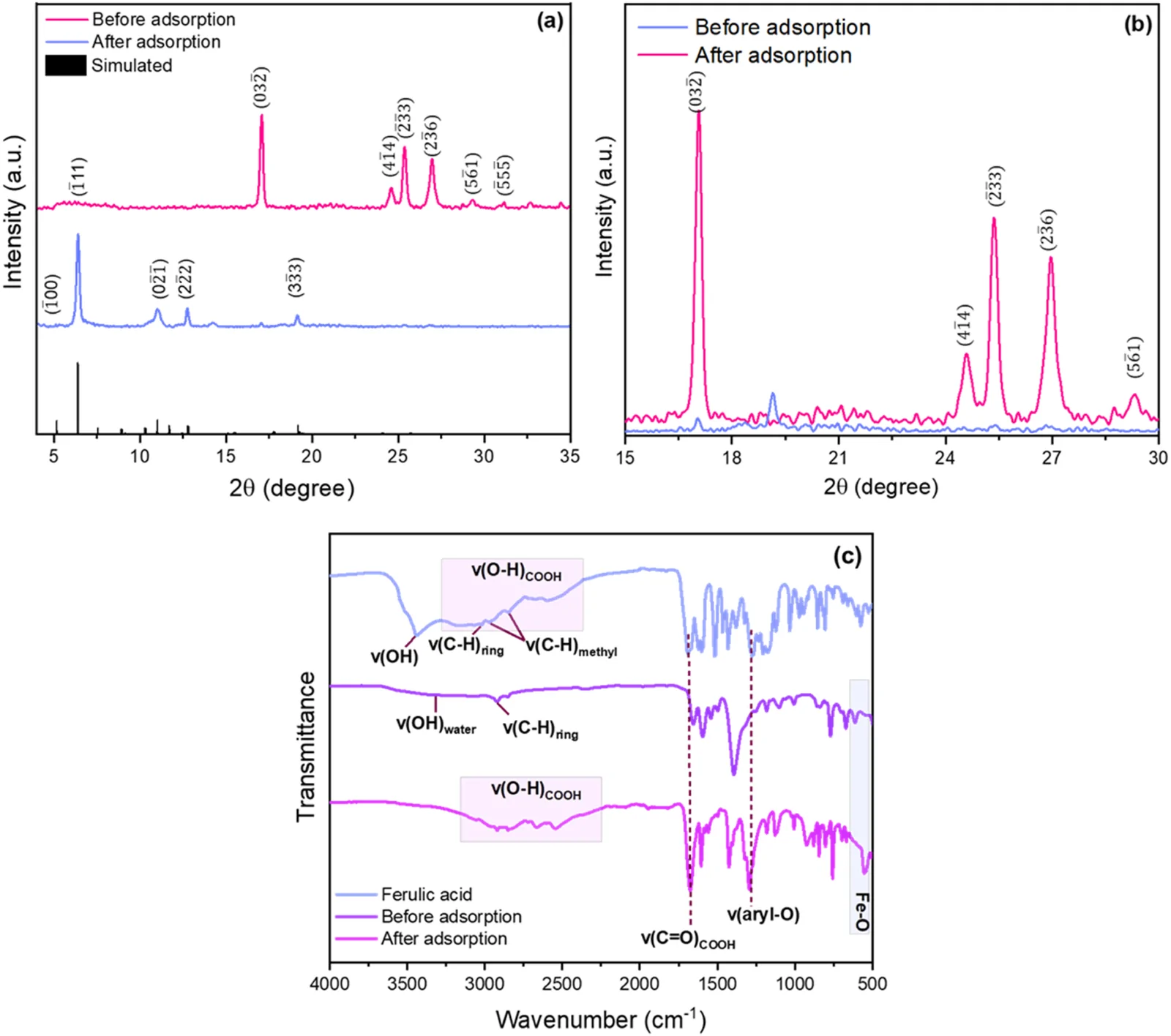
(a) XRD patterns before and after FA adsorption compared with the simulated MIL-88D(Fe) pattern, (b) magnified diffraction region, and (c) FTIR spectra of FA, pristine MIL-88D(Fe), and FA-loaded MIL-88D(Fe).

Consistent with the diffractograms in Fig. 9a, the FT-IR spectra (Fig. 9c) show that MIL-88D(Fe) exhibits a shift in the vibrational mode corresponding to Fe–O (600–500 cm^−1^) before and after adsorption.^23,53^ This behavior could be attributed to the breathing effect induced by guest adsorption. FA exhibits a band around ∼3435 cm^−1^ attributed to the phenolic *ν*(O–H) stretching vibration, a signal at ∼3019 cm^−1^ corresponding to aromatic *ν*(C–H) stretching, and bands at ∼2969 and ∼2841 cm^−1^ assigned to the *ν*(C–H) stretching vibrations of the methyl group. In addition, the broad band between ∼3300 and ∼2300 cm^−1^ corresponding to the *ν*(O–H) of the carboxylic acid and the bands at ∼1692 cm^−1^ and 1290 cm^−1^ attributed to *ν*(C=O) and *ν*(aryl–O) stretching, respectively are still observed after adsorption. Finally, the disappearance of the phenolic *ν*(O–H) and aromatic *ν*(C–H) bands, along with the retention of the carboxylic acid *ν*(O–H), *ν*(C=O), and *ν*(aryl–O) bands, suggests hydrogen-bonding and π–π interactions between FA and MIL-88D(Fe), ruling out direct coordination and the formation of ferulate species.^54,55^

Therefore, the capacity of MIL-88D(Fe) may reflect a more balanced and potentially reversible adsorption process. This characteristic is highly advantageous for applications focused on the cyclic recovery and valorization of FA, as it could facilitate more efficient desorption and adsorbent regeneration, ultimately improving the process's economic feasibility and sustainability.

The diffraction results evidence the reversible lp-to-np breathing transition upon FA uptake, whereas FT-IR confirms that adsorption occurs mainly through hydrogen bonding and π–π interactions without formation of ferulate species.

The achieved removal efficiency validates the potential of this MOF for the valorization of agricultural waste streams. Rather than being lost during conventional wastewater treatment, FA can be efficiently recovered using MIL-88D(Fe) for subsequent applications in the food and pharmaceutical industries, owing to its well-established antioxidant properties.

Pyrzynska *et al.*, highlighted that the use of solid sorbents for FA recovery and purification can be economically challenging at an industrial scale.^1^ In this context, MOFs offer additional functional advantages beyond adsorption. Fe-based MOFs have been identified as promising platforms not only for recovery but also for the encapsulation and controlled delivery of bioactive compounds.^56^ This dual functionality opens the possibility of integrating recovery and formulation processes, where the adsorbed FA could be directly utilized without requiring complete desorption, thereby reducing processing steps and overall costs.

### 3.6. Stability and reusability

The recyclability of MIL-88D (Fe) was evaluated over five consecutive adsorption–desorption cycles under the optimized experimental conditions. As shown in Fig. 10, the FA removal efficiency gradually decreased from 87.19% in the first cycle to 85.30, 84.41, 82.69, and 75.19% in cycles 2–5, respectively. After five cycles, the material retained approximately 86.24% of its initial removal efficiency, indicating that MIL-88D(Fe) can be regenerated and reused while maintaining substantial adsorption performance. The gradual decrease in removal efficiency may result from incomplete desorption of FA, progressive occupation or blockage of adsorption sites, adsorbent losses during repeated recovery and washing steps, or partial changes in the accessibility of adsorption domains. A moderate decline in performance over repeated regeneration cycles is commonly observed for MOF-based adsorbents and does not necessarily imply complete framework degradation. Regenerability and structural stability are nevertheless critical factors for assessing the practical implementation of MOFs in water treatment.^42,57^

**Fig. 10:**
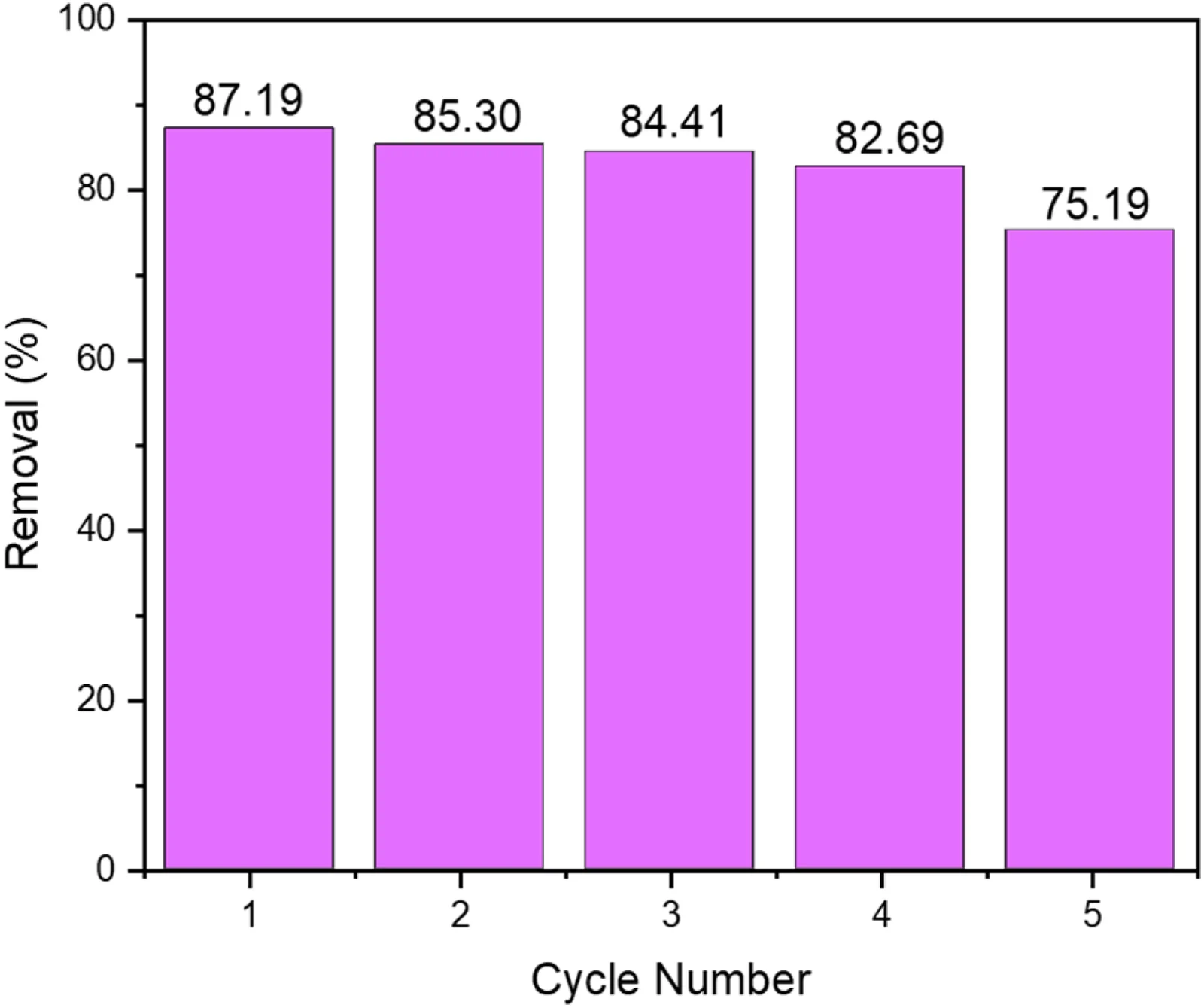
Reusability of MIL-88D(Fe) over five consecutive FA adsorption–desorption cycles. Removal efficiencies for cycles 1–5 was 87.19, 85.30, 84.41, 82.69, and 75.19%, respectively.

To confirm the structural integrity and morphological stability of the adsorbent, powder X-ray diffraction (PXRD) and scanning electron microscopy (SEM) analyses were performed (Fig. 11). The PXRD pattern of pristine MIL-88D(Fe) exhibited a characteristic sharp peak at approximately 2*θ* = 6.5° (Fig. 11a). Upon FA adsorption, this peak shifted or disappeared while new reflections emerged at higher angles, which is characteristic of the structural flexibility (“breathing” effect) commonly observed in MIL-family MOFs during guest molecule entrapment. Remarkably, after the desorption process, the original diffraction peak at 2*θ* = 6.5° completely re-emerged, and the adsorption-induced peaks vanished, confirming a reversible crystalline transition without structural degradation. Furthermore, SEM micrographs taken after the desorption process revealed that the typical rod-like morphology of MIL-88D(Fe) remained completely intact; individual crystals with lengths of approximately 920–950 nm were clearly identified (Fig. 11b), preserving the overall microcrystalline framework and showing no signs of collapse or agglomeration (Fig. 11c). These results collectively demonstrate that MIL-88D(Fe) possesses exceptional crystalline stability and morphological robustness during the cyclic regeneration processes.

**Fig. 11:**
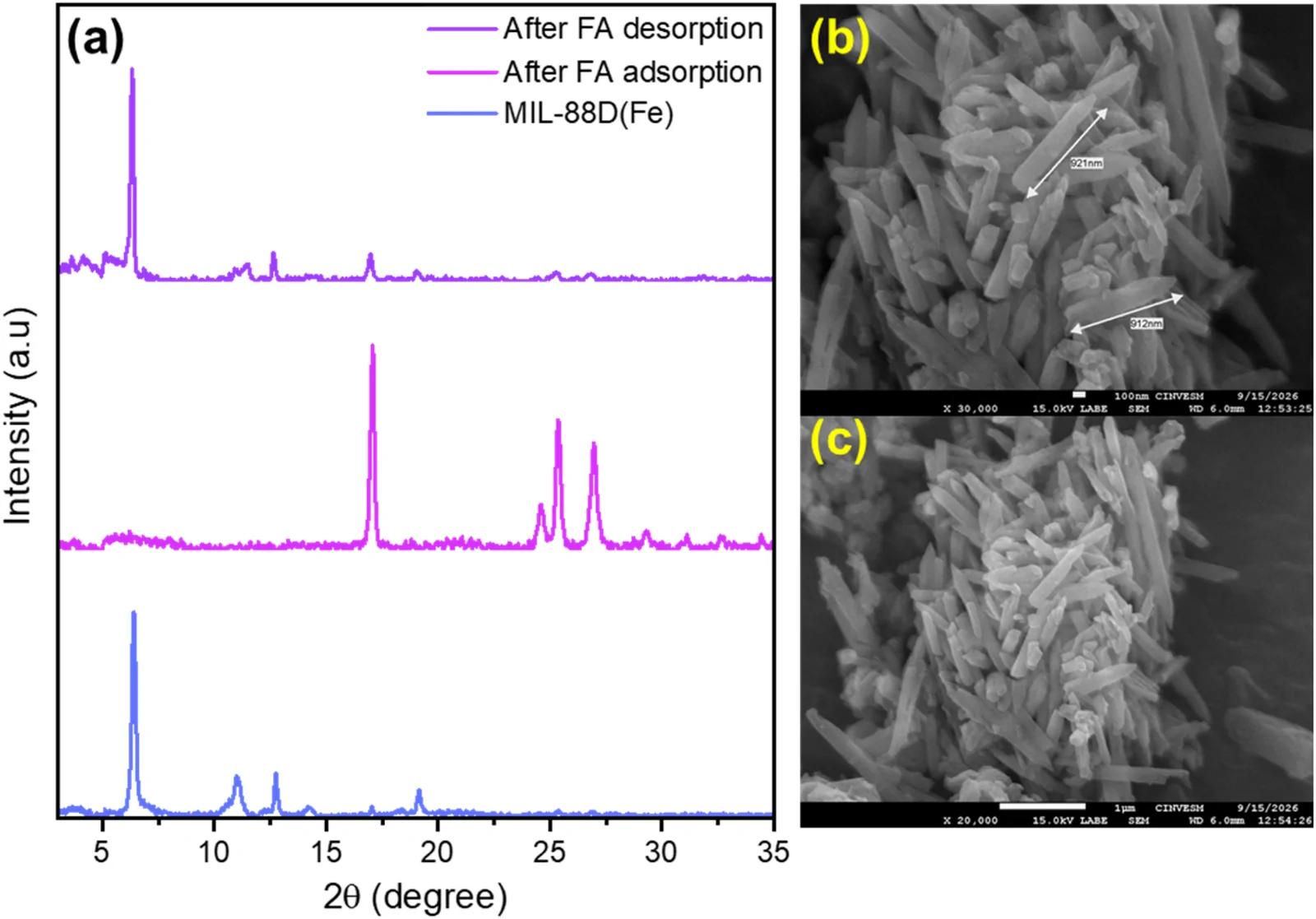
Structural and morphological characterization: (a) PXRD patterns of pristine MIL-88D(Fe), after FA adsorption, and after FA desorption; (b and c) SEM micrographs of the MIL-88D(Fe) framework after FA desorption at different magnifications.

## 4. Conclusions

This study establishes a proof-of-concept for the recovery, rather than only the removal, of ferulic acid from nixtamalization wastewater using the flexible iron-based MOF MIL-88D(Fe). The most significant outcome is the ability of the material to operate in a chemically complex real effluent while allowing subsequent alkaline recovery of FA and repeated adsorbent reuse. The preservation of crystallinity and rod-like morphology after regeneration, together with the retention of 86.24% of the initial removal performance after five adsorption–desorption cycles, indicates that the structural flexibility of MIL-88D(Fe) is compatible with reversible guest uptake without irreversible framework collapse. These findings expand the potential role of flexible MOFs from pollutant removal toward recovery-oriented treatment of agro-industrial effluents containing valuable bioactive molecules. Nevertheless, the present results should be regarded as a laboratory-scale demonstration rather than evidence of industrial or economic feasibility. Further work should address longer-term cycling, batch-to-batch variability of real nejayote, continuous-flow operation, possible iron leaching, and techno-economic and life-cycle assessments to determine the feasibility of scaling this approach for circular-economy applications.

## Author contributions

W. Y. Padrón-Hernández contributed to the synthesis of the materials and the characterization by SEM and BET, interpretation of the results and writing of the manuscript. L. M. Cervera-Andrade, contributed to the synthesis of the materials and doing all the experiments and writing of the manuscript. A. P. M.-Hernández-Muñoz contributed to the characterization of the materials by FTIR and XRD, calculation of kinetic parameters and adsorption isotherms. D. D. Cabañas-Vargas contributed to the interpretation of the results and writing of the manuscript. E.G. Uc-Cayetano, to the interpretation of the adsorption results, interpretation of the results, calculation of kinetic parameters and adsorption isotherms. Geonel Rodríguez-Gattorno contributed to the interpretation of the results, led the research team, and writing of the manuscript. M. N. Sánchez-González contributed to the interpretation of the results, led the research team, and writing of the manuscript. M. A. Estrella-Gutiérrez contributed to the interpretation of the results, led the research team, funding, and writing of the manuscript. All authors discussed the results and contributed to the final manuscript.

## Conflicts of interest

The authors declare that they have no known competing financial interests or personal relationships that could have appeared to influence the work reported in this paper.

## Data Availability

The data supporting the findings of this study are available within the article file. Additional data that support the findings of this study are available from the corresponding authors upon reasonable request.
